# Supplementary material for: Effects of Trichoderma harzianum combined with Phanerochaete chrysosporium on lignin degradation and humification during chicken manure and rice husk composting
Source: Front Microbiol. 2025 Feb 28;16:1515931. doi: 10.3389/fmicb.2025.1515931 (PMC11906335; doi:10.3389/fmicb.2025.1515931)
Supplement: Supplementary file 1 [file Data_Sheet_1.zip › 6.Function/All/FunGuild/l2/Ternary/HS_BFS_CK0_Ternary.pdf]

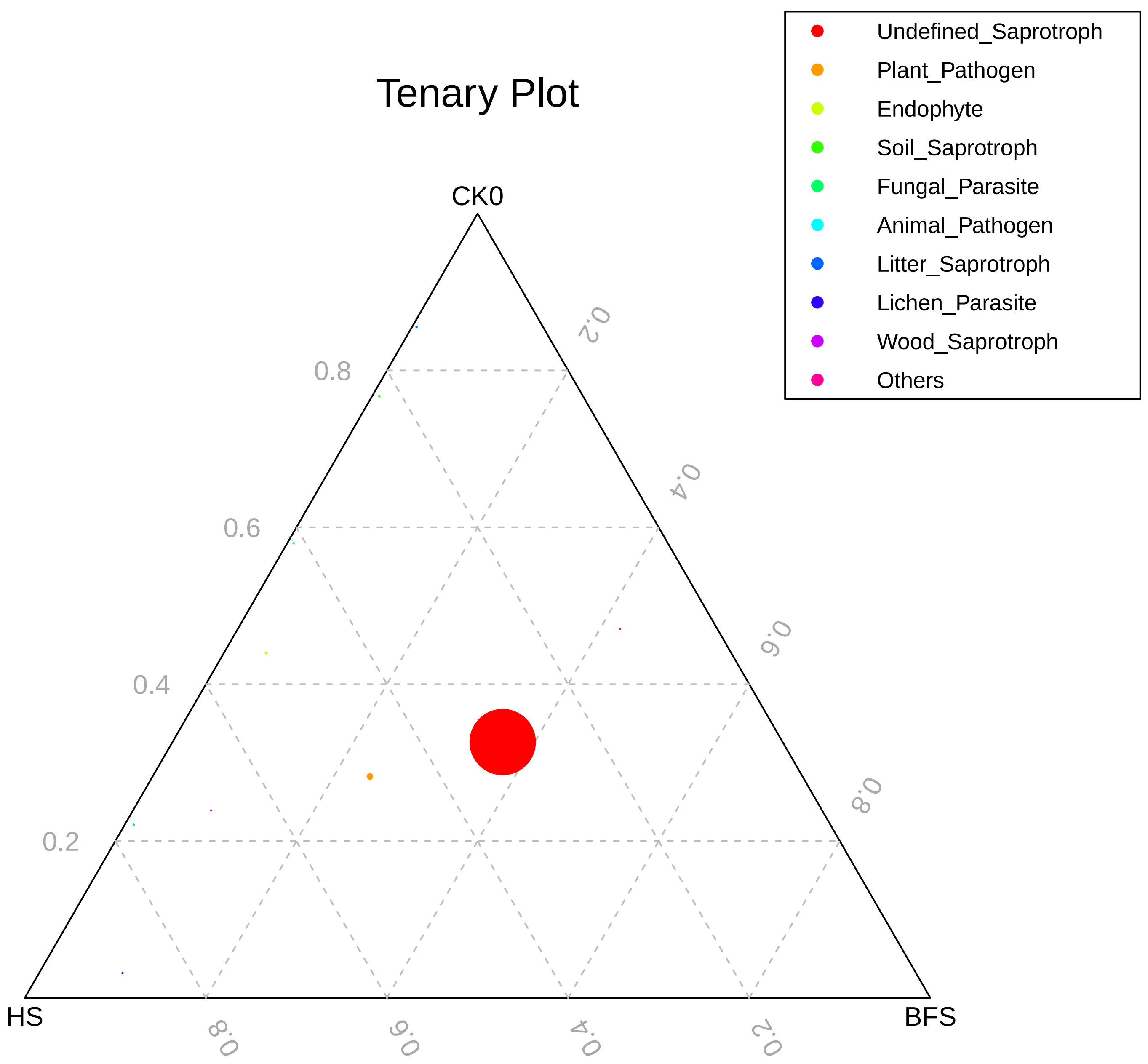
Tenary Plot
CK0
HS
BFS
0.2
0.4
0.6
0.8
Undefined_Saprotroph
Plant_Pathogen
Endophyte
Soil_Saprotroph
Fungal_Parasite
Animal_Pathogen
Litter_Saprotroph
Lichen_Parasite
Wood_Saprotroph
Others
